# Supplementary figures and images for: The skin is a significant but overlooked anatomical reservoir for vector-borne African trypanosomes
Source: eLife. 2016 Sep 22;5:e17716. doi: 10.7554/eLife.17716 (PMC5065312; doi:10.7554/eLife.17716)

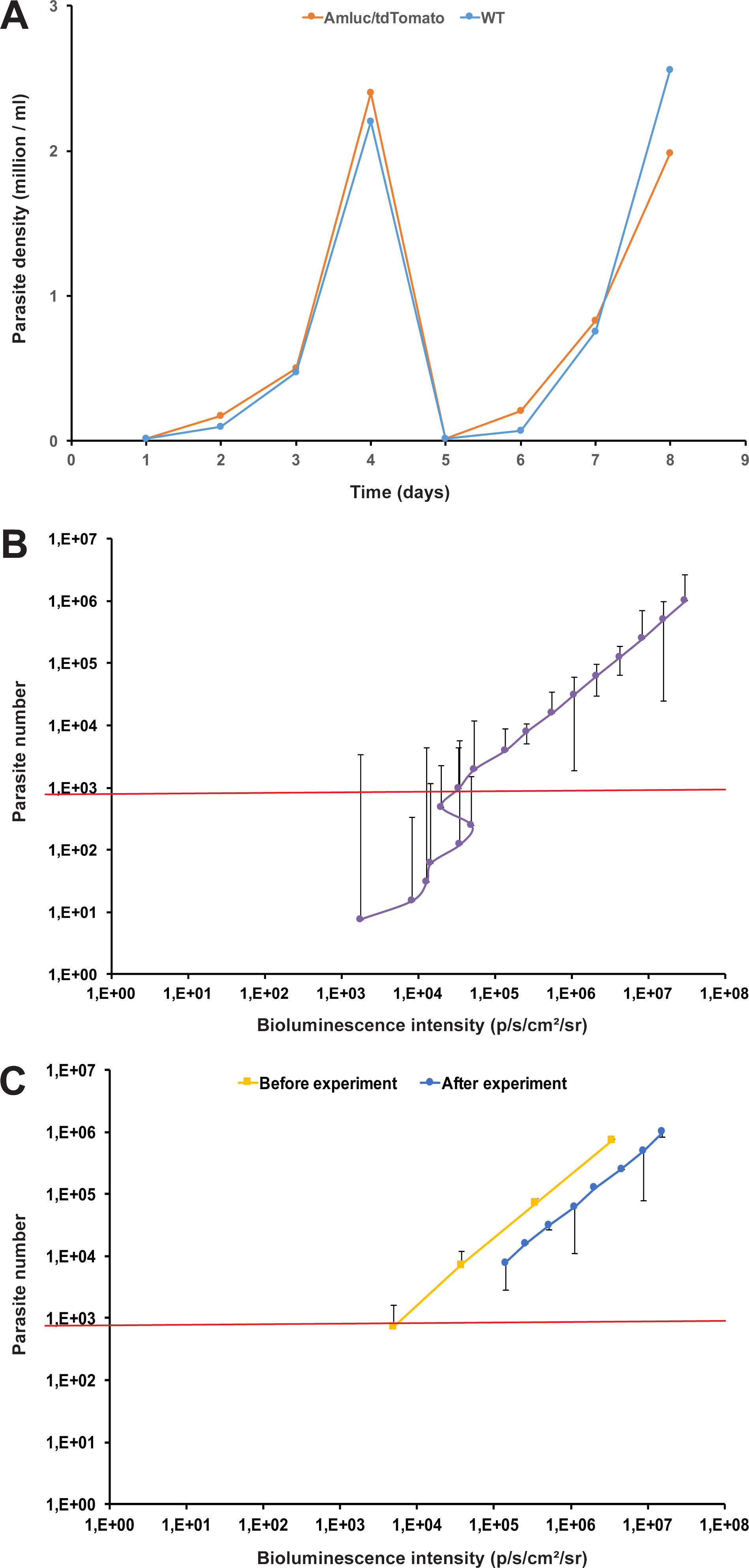

Supplement: Table 1—source data 1. — (A) The in vitro growth of the selected AnTat1.1E AMLuc/TY1/tdTomato sub-clone (red) was similar to that of the parental wild-type strain (blue). Bloodstream forms were cultured in HMI11, counted daily in a Muse cytometer (Merck-Millipore) and diluted after 4 days. (B) A parasite density / bioluminescence intensity analysis was performed by measuring the bioluminescence in successive 2-fold dilutions in 96-micro-well plates with an IVIS Spectrum imager (Perkin Elmer). When plotted as mean ± SD (n = 3), parasite densities and bioluminescence intensities were correlated when the bioluminescence levels were higher than 10 (Berthier et al., 2016) p/s/cm²/sr, corresponding to about 10 (Koffi et al., 2006) parasites, allowing estimation of the parasite density from in vivo imaging over this threshold. This standard curve was used to estimate the number of parasites in the skin from measured values of bioluminescence. (C) This correlation was verified by quantification in a microplate reader Infinite 200 (Tecan) at the very beginning of the first in vivo experiment as well as the end of the last one (mean ± SD, n = 3). DOI: http://dx.doi.org/10.7554/eLife.17716.017 [file elife-17716-table1-data1.jpg]

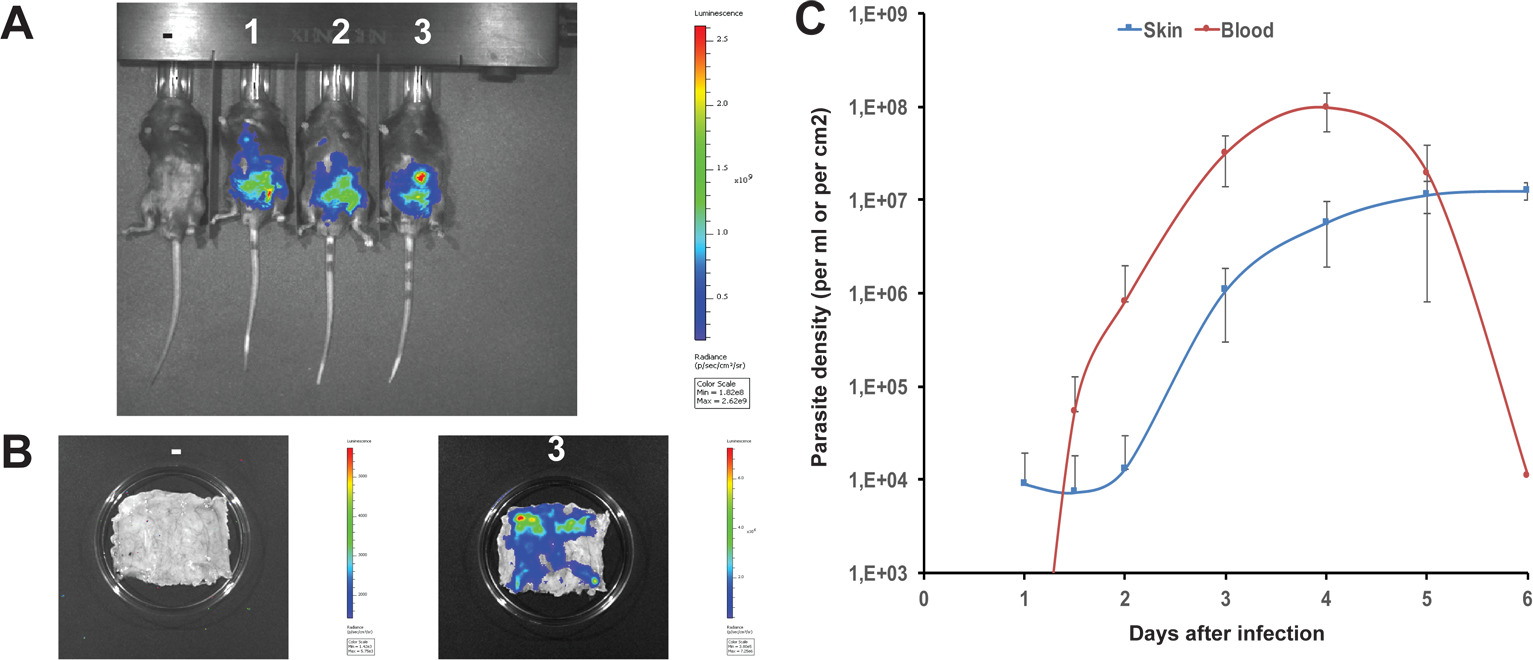

Supplement: Table 1—source data 2. — Mice were injected IP with AnTat1.1E AMLuc/TY1/tdTomato and monitored daily for bioluminescence and parasitaemia. (A) Bioluminescence profile of four mice (- uninfected control and (1–3) three infected mice) four days after infection. (B) The entire skins of the uninfected control mouse (-) and mouse 3 were dissected for bioluminescence imaging four days after infection. (C) Parasite densities in the blood and in the skin (calculated from the mean dorsal bioluminescence intensity measurement and from the standard curve in Table 1—source data 1, in parasites/cm (Fakhar, 2013) in blue) were calculated daily over one week and plotted as mean ± SD (n = 13 mice). DOI: http://dx.doi.org/10.7554/eLife.17716.018 [file elife-17716-table1-data2.jpg]

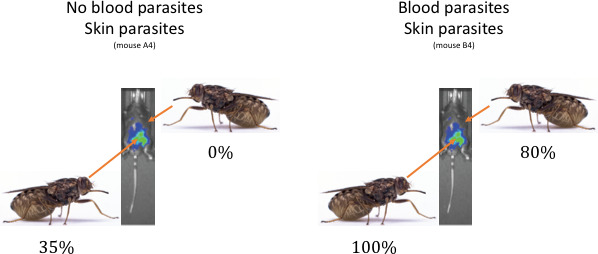

Supplement: Table 1—source data 3. — Schematics summarising the principal results from the xenodiagnosis experiment. In a mouse with no detected transmissible parasites in the blood (absence of stumpy forms by IFA and absence of infection of flies fed on a non-bioluminescent region of the skin), flies can ingest transmissible parasites from the bioluminescent region of the skin (left panel). When a mouse presents transmissible forms in the blood, fly infection rates increase with the concomitant ingestion of parasites from the skin (right panel). Values correspond to those obtained for mouse A4 and B4. DOI: http://dx.doi.org/10.7554/eLife.17716.019 [file elife-17716-table1-data3.jpg]

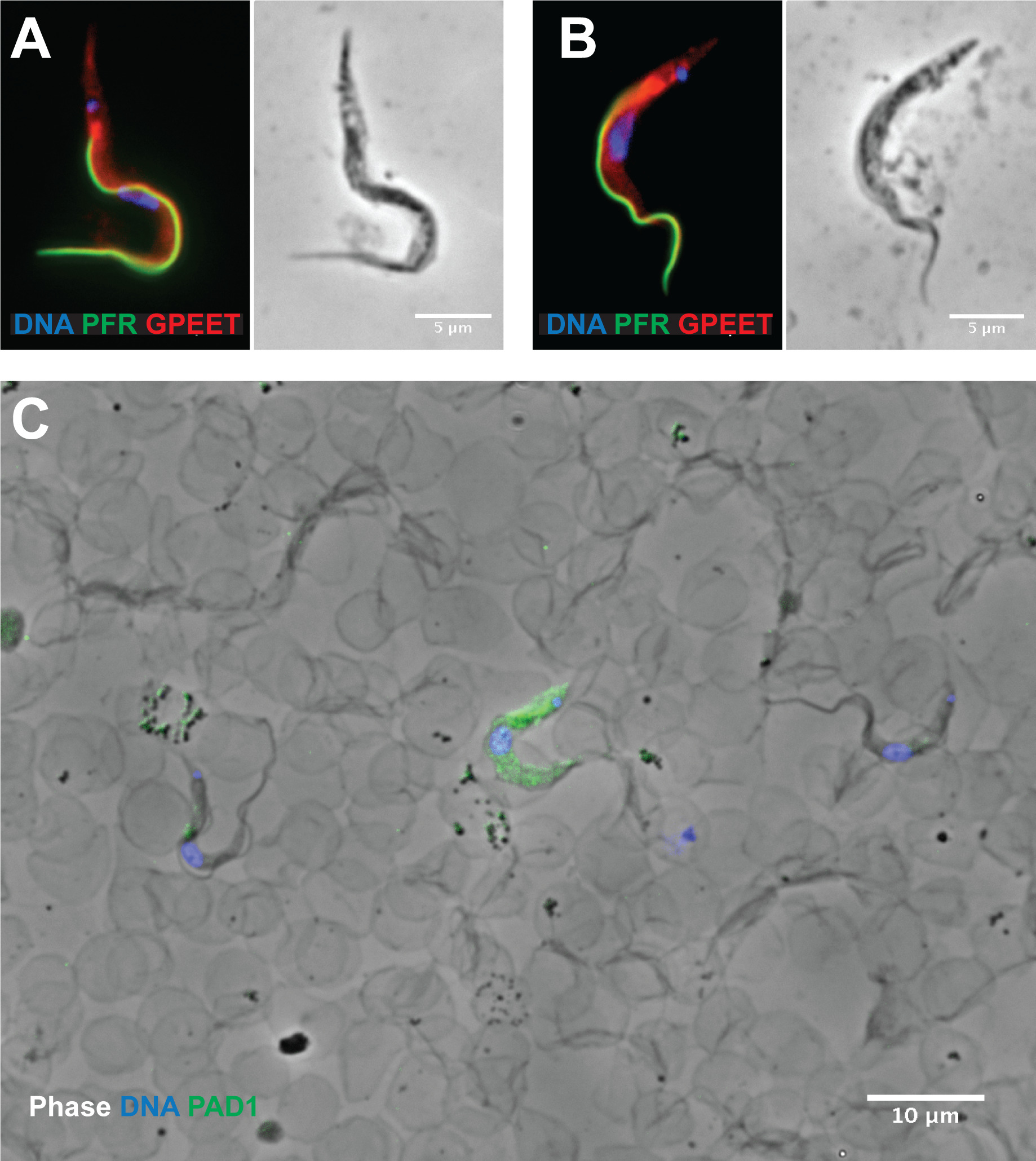

Supplement: Table 1—source data 4. — Parasites recovered from infected tsetse midguts (A–B) or included in bloodsmears (C) were fixed in methanol for 5 s and stained either with the anti-GPEET antibody detecting early procyclic forms (red in A–B) and the L8C4 antibody labelling the flagellum PFR (green in A–B), or with the anti-PAD1 antibody detecting intermediate and stumpy forms (green in C), respectively. DOI: http://dx.doi.org/10.7554/eLife.17716.020 [file elife-17716-table1-data4.jpg]
